# Clinical, Epidemiological, Morphological, and Immunohistochemical Aspects of Nasopharyngeal Carcinoma—4-Year Retrospective Study in the Western Part of Romania

**DOI:** 10.3390/diagnostics14070722

**Published:** 2024-03-29

**Authors:** Maria Alina Marin, Raluca-Maria Closca, Aurel Marin, Marina Rakitovan, Adrian Nicoara, Marioara Poenaru, Marius Militaru, Flavia Baderca

**Affiliations:** 1 ENT Department, University of Medicine and Pharmacy “Victor Babes”, 300041 Timisoara, Romania; alina.marin@umft.ro (M.A.M.); poenaru.marioara@umft.ro (M.P.); 2 ENT Department, Emergency City Hospital, 400139 Cluj-Napoca, Romania; 3 Department of Microscopic Morphology, University of Medicine and Pharmacy “Victor Babes”, 300041 Timisoara, Romania; marina.rakitovan@umft.ro (M.R.); baderca.flavia@umft.ro (F.B.); 4 Department of Pathology, Emergency City Hospital, 300254 Timisoara, Romania; 5 ENT Department, Emergency Pediatric Hospital, 400001 Cluj-Napoca, Romania; skinsens@yahoo.com; 6 Oro-Maxillo-Facial Surgery Clinic, Emergency City Hospital, 300062 Timisoara, Romania; nicoara.adrian@umft.ro; 7 Discipline of Dentoalveolar Surgery, University of Medicine and Pharmacy “Victor Babes”, 300041 Timisoara, Romania; 8 ENT Department, Emergency City Hospital, 300254 Timisoara, Romania; 9 Department of Neuroscience, Discipline of Neurology II, University of Medicine and Pharmacy “Victor Babes”, 300041 Timisoara, Romania; marius.militaru@umft.ro

**Keywords:** head and neck, nasopharyngeal carcinoma, undifferentiated carcinoma, squamous cell carcinoma, immunohistochemistry, combination approaches

## Abstract

Nasopharyngeal carcinoma is one of the most common malignant tumors in the head and neck region. The carcinogenesis is a complex process stimulated by many factors. Although the etiological factors and pathogenic mechanisms are not elucidated, the genetic susceptibility, environmental factors, and association with latent infection with Epstein–Barr Virus play an important role. The aim of this study was to present the main clinical and epidemiological data, as well as the morphological aspects and the immunohistochemical profile, of patients with nasopharyngeal carcinoma diagnosed in western Romania. The study was retrospective and included 36 nasopharyngeal carcinomas. The histopathological diagnosis was completed using immunohistochemical reactions for the following antibodies: p63, p53 and p16 protein, cytokeratins (CK) AE1/AE3, CK5, CK7, CK20 and 34βE12, epithelial membrane antigen (EMA), Epstein–Barr virus (EBV), leukocyte common antigen (LCA), CD20, CD4, CD8, CD68, CD117, and CD1a. The squamous malignant component of nasopharyngeal carcinoma presented with positivity for cytokeratins AE1/AE3, CK5, 34βE12, and p63. Undifferentiated nasopharyngeal carcinoma was positive for EMA in 67% of cases, and 28% of cases showed an immunoreaction for CD117 in the malignant epithelial component. Also, the p53 protein was positive in all the cases. One case of undifferentiated nasopharyngeal carcinoma was p16-positive, and two cases were positive for EBV. A peri- and intratumor cellular infiltrate rich in lymphocytes, with a predominance of CD20-positive B lymphocytes, interspersed with T lymphocytes, was observed. The T cells were CD4- and CD8-positive, predominantly intratumoral, and the CD4:CD8 ratio was 1:1 for 75% of the undifferentiated subtype and 89% for differentiated non-keratinized squamous cell carcinoma. All subtypes of nasopharyngeal carcinoma presented with an inflammatory infiltrate with numerous plasma cells, eosinophils, and dendritic cells, presenting as antigen CD1a- and CD68-positive, as well as in CD117-positive mast cells.

## 1. Introduction

Nasopharyngeal carcinoma is an epithelial carcinoma occurring from the nasopharyngeal mucosa [1]. The pharyngeal recess (fossa of Rosenmuller) is the most common affected site, followed by the superior posterior wall of the nasopharynx [2]. Although it accounts for only 0.7% of all malignant tumors, it represents one of the most common malignancies in the head and neck region [3]. The rate of incidence increases from ages 20 to around 50 [4]. It occurs more often in men, with the incidence being 2.5 times higher in men than in women [5].

Nasopharyngeal carcinoma is highly associated with latent infection with Epstein–Barr virus. It has a characterized geographical distribution, and it is endemic in a few areas, including Southeast Asia, Southern China, North Africa, and the Artic. In Southern China, it represents a major cause of morbidity and mortality [6].

The underlying mechanisms behind this geographic distribution have not been elucidated. Although Epstein–Barr virus infection has been suggested as a necessary cause of undifferentiated nasopharyngeal carcinoma, the virus itself is not sufficient to cause this malignancy; other co-factors, such as environmental and genetic factors, may interact with Epstein–Barr virus to play a role in the pathogenesis of this tumor. Environmental exposures, such as the high consumption of salt-preserved fish, tobacco smoking, lack of fresh fruit and vegetable intake, formaldehyde, and wood dust, are factors involved in the carcinogenesis of nasopharyngeal carcinoma [7].

Most patients present with a locoregionally advanced stage, due to its deep location and lack of obvious clinical signs at an early stage. The clinical presentation relates to the presence of a mass in the nasopharynx area (epistaxis, obstruction, and blood-stained postnasal drip), also affecting the Eustachian tube (hearing impairment, serous otitis media, and tinnitus), skull base involvement with cranial nerve impairment (headache, diplopia, facial pain, numbness, or paresthesia), and a painless neck mass due to metastasis of the regional lymph nodes; 5% of cases show distant metastasis at the initial presentation, while 10% of patients are asymptomatic [8].

No laboratory blood test provides high sensitivity or specificity for the diagnosis of nasopharyngeal carcinoma. Magnetic resonance imaging of the nasopharynx and cervical region is the imaging modality of choice to assess the extent of disease and the presence of intracranial extension. Nasopharyngeal endoscopy accompanied by biopsy and a microscopic exam of suspected tissues remains the gold standard for the diagnosis of nasopharyngeal carcinoma [9].

The World Health Organization has classified nasopharyngeal carcinoma into three histological types: keratinizing squamous cell carcinoma, non-keratinizing squamous cell carcinoma, and basaloid squamous cell carcinoma, based on the tumor cell appearance under the light microscope. The non-keratinizing type is subdivided into differentiated non-keratinizing carcinoma and undifferentiated carcinoma and is predominantly Epstein–Barr-virus-related [10].

Based on a microscopic examination, the keratinizing squamous cell carcinoma shows squamous differentiation at the light-microscopic level, with intercellular bridges and/or various degrees of keratinization. The undifferentiated non-keratinizing squamous cell carcinoma is characterized by large tumor cells with a syncytial appearance, round-to-oval vesicular nuclei, and large central nucleoli. The differentiated subtype of non-keratinizing squamous cell carcinoma exhibits cellular stratification, often with a plexiform pattern, and the neoplastic cells are slightly smaller, with a lower nuclear–cytoplasmic ratio, more chromatin-rich nuclei, and less prominent nucleoli. Focally, intercellular bridges may be present. The irregular islands and sheets of malignant cells are intimately intermingled with variable numbers of lymphocytes and plasma cells. The basaloid squamous cell carcinoma is the rarest subtype and consists of islands of small oval cells, with scant cytoplasm and hyperchromatic nuclei [10].

Differentiated keratinizing carcinoma accounts for less than 20% of all cases worldwide. The association of Epstein–Barr virus with this type has been found particularly in the geographical regions with a high incidence of undifferentiated nasopharyngeal carcinoma. The virus exists in a latent state, exclusively in the tumor cells and absent from the surrounding lymphoid infiltrate. The interaction between the lymphoid stroma found in undifferentiated nasopharyngeal carcinoma and adjacent malignant cells appears to be crucial for the continued growth of the tumor [6].

The most important prognostic factor is the stage at presentation [11]. Thus, the 10-year survival rate for patients with nasopharyngeal carcinoma can reach 98% for stage I and 60% for stage II. In contrast, the median survival is 3 years for patients in the advanced stages of the disease [12].

An increasing tumor volume represents a negative factor, with an estimated 1% increase in risk of local failure per 1 cm^3^ increase in volume. Other unfavorable prognostic factors are fixation of the neck nodes, male sex, age over 40 years, and cranial nerve palsy. The histological subtype is also a prognostic factor. Thus, keratinized carcinomas had worse prognoses than non-keratinizing carcinomas, show a greater propensity for locally advanced tumors, have a lower propensity for lymph node metastasis, and are less responsive to radiation therapy [13].

Radiotherapy is the primary treatment modality for nasopharyngeal carcinoma. Effective curative treatment requires optimal radiotherapy planning, with precise beam delivery that maximizes locoregional control and reduces treatment-related side effects [14]. Although radiation therapy remains the mainstay treatment, types 2 and 3 of nasopharyngeal carcinoma have been shown to be chemosensitive in all stages of the disease [2].

## 2. Aim of the Study

The aim of this study was to present the clinical, epidemiological, morphological, and immunohistochemical aspects of patients with nasopharyngeal carcinoma diagnosed within a period of four years, in the Service of Pathology of the Emergency City Hospital, Timisoara, Romania.

## 3. Patients, Materials, and Methods

The study was retrospective, with a chronological extension over four years, and included cases of nasopharyngeal carcinoma diagnosed in the Service of Pathology of Timisoara’s Emergency City Hospital. The cases were biopsied in the Department of Ear, Nose and Throat Clinic between 1 January 2015 and 21 December 2018.

The cases were identified using the specimen’s reception registers of the Service of Pathology and were integrated in the clinical context using the computer database of Timisoara’s Emergency City Hospital.

The inclusion criteria were as follows: age at onset over 18 years, nasopharyngeal carcinoma with a definite histopathological diagnosis, nasopharyngeal squamous cell carcinoma, nasopharyngeal differentiated keratinized squamous cell carcinoma, nasopharyngeal differentiated non-keratinized squamous cell carcinoma, undifferentiated nasopharyngeal carcinoma, primary nasopharyngeal tumor, specimens resulting from incisional biopsy of the nasopharyngeal tumor mass or excisional nodal biopsy, tumors with a complete immunohistochemical profile, absence of recurrence, absence of radio- or/and chemotherapy in the head and neck area.

The exclusion criteria from the study were as follows: age at onset under 18 years, secondary carcinoma in the nasopharyngeal area, other histopathological subtypes (mesenchymal tumor, neuroendocrine, or lymphoid tumor), uncertain or incomplete histopathological result, incomplete immunohistochemical profile, recurrent tumor, radio- or/and chemotherapy in the head and neck area.

In order to make this study possible in accordance with the ethical norms imposed by the Declaration of Helsinki, elaborated in 1975 and rectified in 2000, as well as the legislation enforced in Romania, the opinion of the ethics commission of the hospital was obtained. Also, for each case included in the study, we checked the presence of the annex that accompanies the biological samples, which certifies the presence of informed patient consent for the use of histological processed pieces for diagnostic and scientific purposes.

All histopathological slides were processed according to the international protocol adapted to the requirements of the department, in accordance with the recommendations enforced by the Ministry of Health.

Thus, the harvested fragments were fixed in 4% *v*/*v* buffered formaldehyde and processed with the usual histological technique. Four-micrometer-thick sections were cut using a semi-automated Leica RM2235 rotary microtome (Leica Biosystems, Nussloch, Germany), displayed on SuperFrost™ microscope (St. Louis, MO, USA) slides. The slides were stained with a Hematoxylin–Eosin technique in order to obtain the morphological diagnosis. The histopathological diagnosis was completed using immunohistochemical reactions for the following antibodies: anti-cytokeratins AE1/AE3, CK5, CK7, CK20 and CK 34βE12, anti-p63, anti-p53, anti-p16 protein, anti-EMA, anti-LCA, anti-CD20, anti-CD4, anti-CD8, anti-CD68, anti-CD1a, anti-CD117 and anti-EBV (Table 1). All the antibodies and reagents for immunohistochemical reactions were purchased from Leica Biosystems, New Castle, UK.

## 4. Results

### 4.1. Clinical–Epidemiological Findings

**Figure 1:**
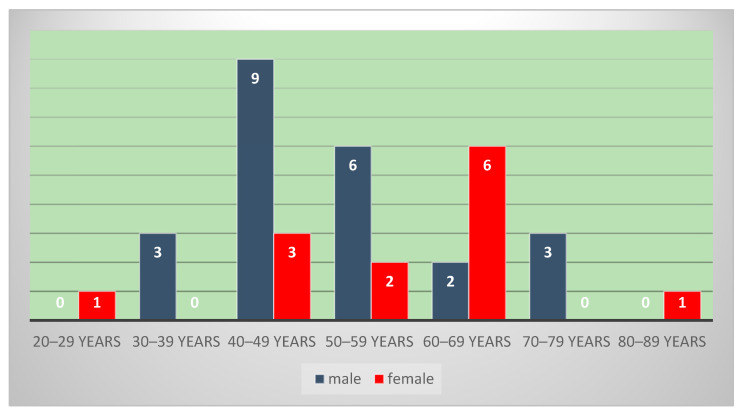
Gender distribution of nasopharyngeal carcinoma according to age, with peak incidence in the 4th decade for males and in the 6th decade for females.

The 36 patients were between 23 and 81 years of age, with an average age of 54 years, and the peak incidence was in the 4th and 5th decade for males (25% respectively 16.6%), and in the 6th decade for female (16.6%). We noticed a predominance of the male sex (63.8%), with a male/female ratio of 1.7 (Figure 1).

**Figure 2:**
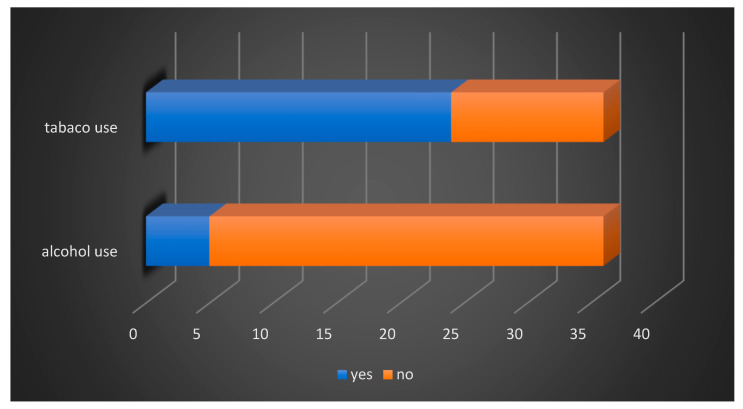
Distribution of patients according to tobacco and alcohol consumption.

Regarding environmental factors, we observed an increased rate of smoking patients (66.6%), while alcohol consumption associated with smoking was present in only five patients (13.8%) (Figure 2).

Most patients presented with onset signs and symptoms related to the presence of the tumor mass in the nasopharynx (75%). Thus, approximately 70% of the patients presented with nasal obstruction, unilateral (27%) or bilateral (41%). Ten of these patients (27%) also associated oral breathing, while nine patients (25%) did not present with symptoms determined by the presence of the tumor mass in the nasopharynx. It was also observed that 39% of patients had signs and symptoms determined based on Eustachian tube dysfunction, and a third of them presented unilateral or bilateral hearing loss. Skull base and cranial nerve involvement was presented in 30% of patients, with headache (25%), diplopia (2.7%), and numbness and paresthesia (2.7%). One patient (2.7%) had nerve II paresis with deviation of the right eyeball (Table 2).

**Figure 3:**
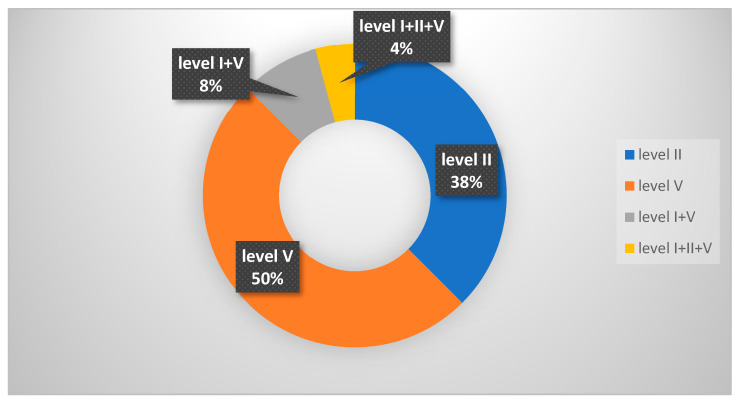
Distribution of cervical lymph node metastasis in nasopharyngeal carcinoma.

Here, 66.6% of patients had cervical adenopathy, and in 25% of cases, this was the initial symptom of onset. It was observed that half of the patients had involvement of the supraclavicular nodes, corresponding to level V, and 38% had the involvement of level II, respectively, the superior jugular nodes (Figure 3).

**Figure 4:**
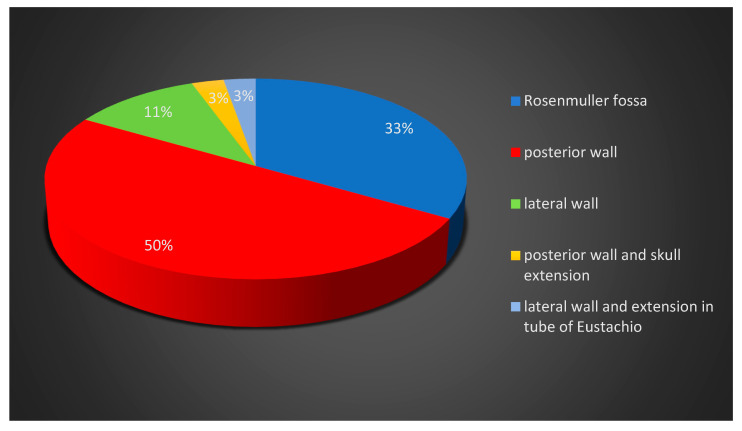
Distribution of patients according to the tumor site.

Due to the signs and symptoms, the patients underwent posterior rhinoscopy and nasal endoscopy, and a presumptive diagnosis of a nasopharyngeal tumor mass was performed. The most common site of origin of nasopharyngeal carcinoma was the posterior wall of the nasopharynx (50%), followed by the Rosenmuller fossa (33%) and the lateral wall of the nasopharynx (11%). In one case, the tumor, localized in the posterior wall of the nasopharynx, presented with extension to the base of the skull, and in another case, the tumor localized in the lateral wall had extension in the retroauricular area (Figure 4).

**Figure 5:**
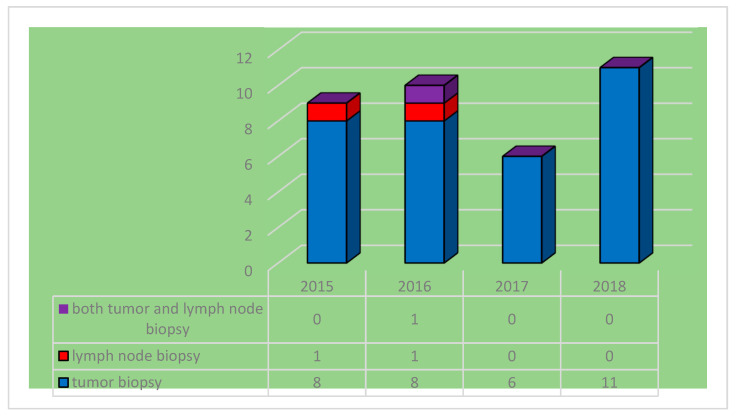
Distribution of biopsy samples by year.

Also, the patients underwent computer tomography or magnetic resonance imaging of the head and neck to evaluate the locoregional tumor extension and for clinical staging. Incisional biopsy of the nasopharynx tumor mass was performed for 33 patients (92%). In two cases (5.5%), only lymph node enlargement was noted at presentation; therefore, the enlarged mass was excised in order to establish the diagnosis. In one patient, both tumor incisional biopsy and lymph node excision were performed (Figure 5).

### 4.2. Histopathological Findings

**Figure 6:**
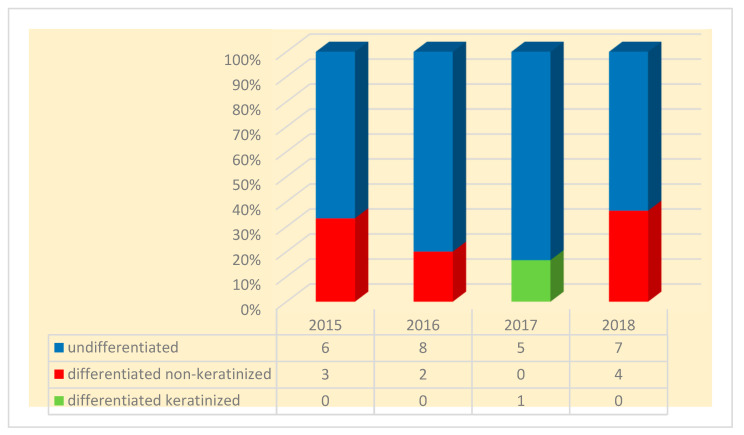
Main histopathological subtype of nasopharyngeal carcinoma.

The microscopic examination revealed, for most cases, an undifferentiated carcinoma of the nasopharynx (72%), while differentiated non-keratinized squamous cell carcinoma was present in 25% of cases. A single case presented with differentiated keratinized squamous cell carcinoma (2.7%) (Figure 6).

**Figure 7:**
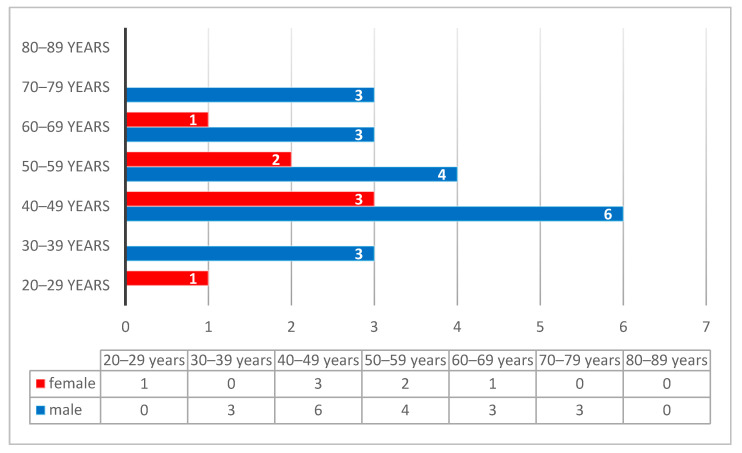
Distribution of undifferentiated nasopharyngeal carcinoma according to decades and gender, with a male predominance and a peak incidence in the 4th decade.

**Figure 8:**
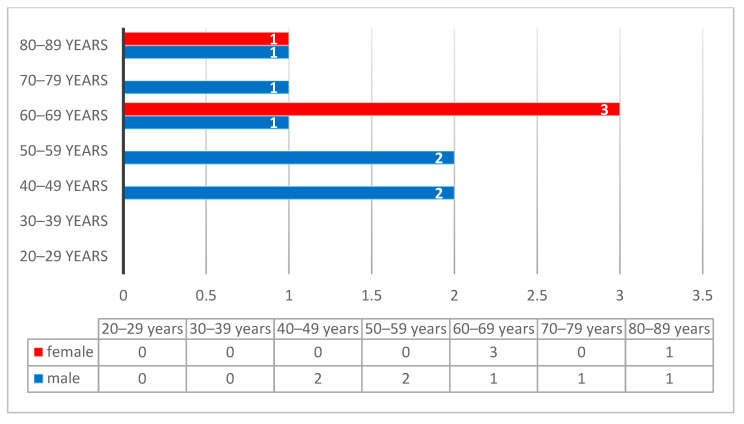
Distribution of the differentiated subtype of non-keratinized squamous cell carcinoma according to decades and gender, with a peak incidence of female in the 6th decade.

The cases diagnosed with undifferentiated nasopharyngeal carcinoma had a male predominance, with a peak incidence in the 4th decade (17%), following by the 5th decade (11%), while the 3rd, 6th, and 7th decades had a rate of 8% each (Figure 7). Unlike undifferentiated carcinoma, the differentiated subtype of non-keratinized squamous cell carcinoma showed an increased incidence in females, with a peak in the 6th decade (Figure 8).

The undifferentiated nasopharyngeal carcinoma showed large tumor cells with a syncytial growth pattern, scant amphophilic or eosinophilic cytoplasm, round-to-oval vesicular nuclei, and large nucleoli. The malignant cells were intermingled with an increased amount of inflammatory infiltrate. The peri- and intratumor inflammatory infiltrate was predominantly composed of lymphocytes, with a variable amount of plasma cells, macrophages, neutrophil granulocytes, and eosinophils. One case was associated with extensive epithelioid necrotizing granuloma, highly suggestive of a mycobacterial etiology.

**Figure 9:**
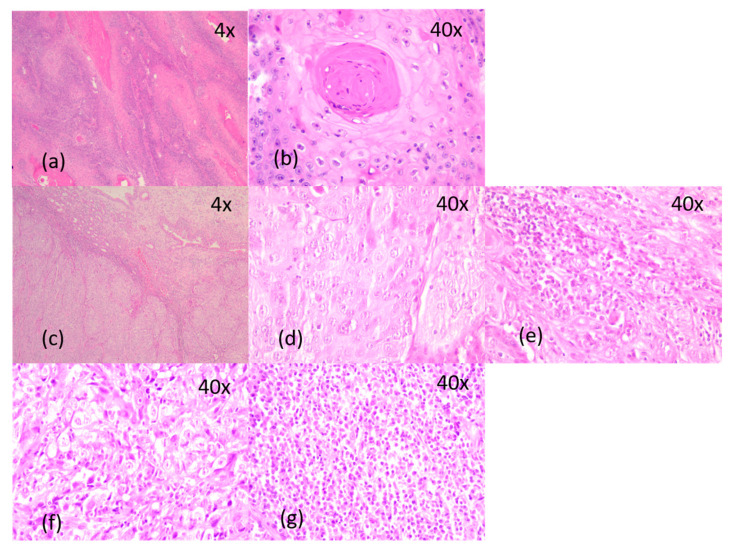
Morphological aspects of the nasopharyngeal carcinoma, hematoxylin–eosin staining: (**a**,**b**) differentiated keratinized squamous cell carcinomas; (**c**–**e**) differentiated non-keratinized squamous cell carcinoma; (**f**,**g**) undifferentiated nasopharyngeal carcinoma.

Keratinizing and non-keratinizing differentiated squamous cell carcinoma showed islands of tumor cells, slightly smaller than those observed in the undifferentiated nasopharyngeal group, with obvious squamous differentiation, intercellular bridges, and various degrees of keratinization, associated with desmoplastic stroma. Unlike undifferentiated carcinoma, in differentiated squamous cell carcinomas, chronic inflammatory infiltrates were absent or reduced (Figure 9).

The histopathological diagnosis was completed using immunohistochemical markers for epithelial/squamous differentiation, for peri- and intratumor immune cellular infiltrates, and for pathogenic and prognostic markers.

Undifferentiated nasopharyngeal carcinoma showed positivity for cytokeratin AE1/AE3, CK5, p63, and high-molecular-weight cytokeratin, with strong reactions in 80–100% of tumor cells. Membrane epithelial antigen was diffusely positive, with moderate/strong reactions in most cases (67%), while two cases were negative. Ten cases (28%) of undifferentiated nasopharyngeal carcinoma showed immunoreactions for CD117 in the malignant epithelial component. Also, the p53 protein was positive in all the cases, with moderate-to-strong immunostaining.

The positive reaction for p16 was identified in a single case of undifferentiated nasopharyngeal carcinoma, being associated with the positive reaction for Epstein–Barr virus. In total, two Epstein–Barr-virus-positive cases were identified.

The immune tumor microenvironment revealed the presence of a peri- and intratumor cellular infiltrate rich in lymphocytes, with a predominance of B lymphocytes, CD20-positive (70–80% of the inflammatory infiltrate), interspersed with T lymphocytes (10–20%). The T cells were CD4-positive T helper lymphocytes, with a peri- and intratumor distribution, and CD8-positive T cytotoxic lymphocytes, predominantly intratumoral. The CD4:CD8 ratio was 1:1 in most cases (75%), respectively, and 2:1 in 25% of cases.

**Figure 10:**
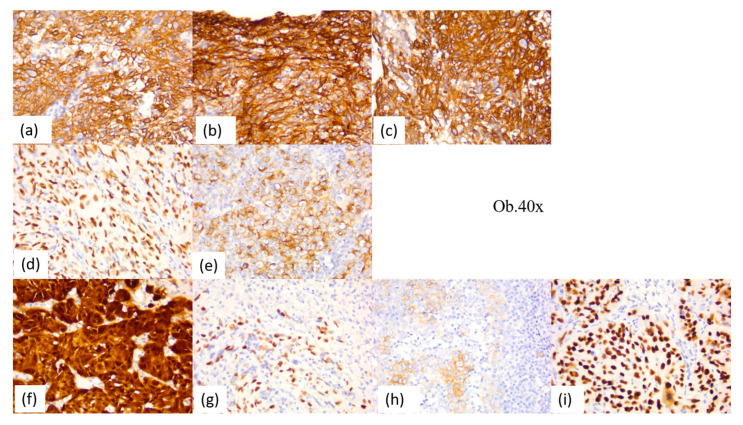
Immunohistochemical aspects of the squamous component of undifferentiated nasopharyngeal carcinoma: (**a**) CK AE1/AE3; (**b**) 34βE12; (**c**) CK5; (**d**) p63; (**e**) EMA; (**f**) p16; (**g**) EBV; (**h**) CD117; (**i**) p53.

**Figure 11:**
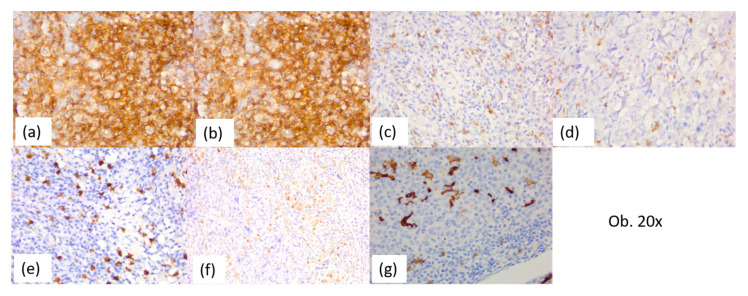
Immunohistochemical aspects of the tumor immune microenvironment of undifferentiated nasopharyngeal carcinoma: (**a**) LCA; (**b**) CD20; (**c**) CD4; (**d**) CD8; (**e**) CD117; (**f**) CD68; (**g**) CD1a.

The inflammatory infiltrate had eosinophils, plasma cells, macrophages, antigen-presenting cells, and CD117-positive mast cells. Thus, all cases of undifferentiated nasopharyngeal carcinoma had up to 5% eosinophils, plasma cells, and mast cells. The positive CD1a and CD68 antigen-presenting cells were observed in most cases of undifferentiated nasopharyngeal carcinoma (88%), being between 5 and 15% of the peri- and intratumor inflammatory infiltrate, with an intratumor predominance. In these cases, the association with macrophages was also observed, with these being up to 1%. It was also noted that three cases (12%) had numerous antigen-presenting cells, predominantly intratumoral, with a percentage between 20 and 30%. The complete immunohistochemical profile of the 26 nasopharyngeal undifferentiated carcinomas can be found in Table 3, and the main immunostaining aspects are detailed in Figure 10 and Figure 11.

The differentiated subtype of keratinizing and non-keratinizing squamous cell carcinoma had no significant differences in the immunohistochemical profile. Thus, they presented with positivity for cytokeratin AE1/AE3, CK5, p63, and high-molecular-weight cytokeratin, with a strong reaction in 80–100% of tumor cells, and membrane epithelial antigen was positive in all cases, with weak-to-moderate immunostaining, while the CD117 reaction were completely negative in the malignant epithelial component. Also, the p53 protein was positive in all the cases, with weak-to-moderate immunostaining. One case of the non-keratinized subtype showed reactivity for p16, with strong and diffuse nuclear and cytoplasmic immunostaining in 100% of tumor cells, and Epstein–Barr virus was not immunohistochemically identified.

The immune tumor microenvironment revealed small amounts of peritumor inflammatory infiltrates, rich in lymphocytes, with B cells predominating, positive for CD20 (70–80% of the inflammatory infiltrate), interspersed with T lymphocytes (10–20%). The T cells were also CD4-positive T helper lymphocytes and CD8-positive T cytotoxic lymphocytes. The CD4:CD8 ratio was 1:1 in most cases (90%), respectively, and 2:1 in one case (10%).

Also, the inflammatory infiltrate had plasma cells, eosinophils, positive CD1a and CD68 antigen-presenting cells, and CD117-positive mast cells. The immunohistochemical profile and the main immunostaining aspects of the 10 cases of the differentiated keratinizing and non-keratinizing squamous cell carcinoma are presented in Table 4.

Most patients with nasopharyngeal carcinoma (97%) were under oncological follow-up and have undergone radiotherapy and/or chemoradiotherapy in accordance with international treatment guidelines. Only one patient refused the oncological treatment.

## 5. Discussion

Nasopharyngeal carcinoma is a squamous cell carcinoma of the head and neck region, with a unique etiopathogenetic mechanism, driven by the interaction of genetic susceptibility and environmental factors, as well as a strong association with the Epstein–Barr virus [15].

Epstein–Barr virus infection is related to the development of cancers originating from both epithelial and lymphoid cells, and 95% of the world’s population has an asymptomatic lifelong infection with the Epstein–Barr virus. This virus persists in the memory B cells of healthy and asymptomatic individuals, and a disruption of this interaction resulting in malignancy. The association of Epstein–Barr virus infection with nasopharyngeal carcinoma is considered to be caused by the aberrant establishment of virus latency in epithelial cells, exhibiting premalignant genetic changes. Despite the fact that the Epstein–Barr virus role in carcinogenesis is poorly known, the identification of the virus in all tumor cells provides opportunities for the development of new diagnostic and therapeutic approaches [1]. The role of the Epstein–Barr virus in the tumorigenesis of nasopharyngeal carcinoma continues to be studied and offers a perspective on the development of targeted therapies for this type of cancer. Novel therapeutic approaches using virus reactivation, gene therapy, and therapeutic vaccination are needed. Also, in high-prevalence regions, screening programs to classify patients with early-stage Epstein–Barr virus-associated carcinoma are already underway [16,17].

The tumorigenesis of nasopharyngeal carcinoma is a complex process associated with numerous factors. Epstein–Barr virus is a causative factor of nasopharyngeal carcinoma and is most likely involved in multistage and multifactorial carcinogenesis. Epstein–Barr-virus-encoded genes have been shown to be involved in immune evasion and the regulation of various cell signaling cascades [4]. Mechanisms underlying carcinogenesis include epigenetic changes, the mutation of genetic codes, chromosome stability, DNA repair, and the process of cell growth. The dysregulation of oncogenes and tumor suppressor genes driven by genetics and epigenetics is considered a driving force in cancer growth and progression. Thus, overexpression of the oncoprotein c-Myc plays a role in the malfunction of several important cellular processes, including the control of cell growth, proliferation, apoptosis, and cell metabolism. Also, p53 protein dysfunction promotes cell proliferation with severe DNA damage and is implicated in the disruption of p53-dependent cell cycle arrest or apoptosis. Epstein–Barr virus expresses viral oncogenes that can determine epigenetic changes and genetic mutations, leading to tumorigenesis, as well as cancer progression [8].

Although the mechanisms of carcinogenesis are less understood, Young and Dawson propose a model in which the loss of heterozygosity occurs early in the pathogenesis of nasopharyngeal carcinoma, probably as a result of relationships with environmental factors, such as dietary factors. It results in preinvasive lesions that, after additional genetic and epigenetic events, become susceptible to stable Epstein–Barr virus infection. Once cells have been infected, latent Epstein–Barr virus genes provide growth and survival benefits, leading to the development of malignancy. Also, additional genetic and epigenetic changes can occur after virus infection [6].

The association with the Epstein–Barr virus and the proximity of tumor cells to the lymphoid structures of the nasopharynx leads to a characteristic tumor immune microenvironment consisting of T lymphocytes, B lymphocytes, macrophages, and dendritic cells [18].

The tumor microenvironment has a dominant role in the progress and invasiveness of the tumor and includes immune and stromal cells, blood vessels, and extracellular matrix, some of them being the target of individualized therapies [19,20].

The tumor immune microenvironment is an important component of the large tumor microenvironment and plays an important role in supporting tumor growth and progression. It has two functional categories of cells: immune-stimulating cells, which facilitate the anti-cancer immune response and include tumor-infiltrating lymphocytes, natural killer cells, and eosinophils, and immunosuppressive cells, which inhibit the anti-cancer immune response to promote tumor progression and include mast cells, regulatory T cells, and macrophages [21]. The balance of immune-stimulating and immunosuppressive cells can prevent or promote tumor progression [22].

The recruitment of immune cells is mediated by the increased expression of chemokines by tumor cells, macrophages, and dendritic cells [23,24]. Therefore, the increased secretion of chemokines from tumor cells facilitates the recruitment of immune cells, and macrophages co-express anti-inflammatory M1 and pro-inflammatory M2 gene signatures, forming an intermediate phenotype that secretes a high level of chemokines and recruits immune cells [18,25]. Also, the macrophages can differentiate from monocytes and secrete chemokines to recruit immune cells. Dendritic cells express programmed death ligand 1 and the ligand for cytotoxic T-lymphocyte-associated protein 4, inducing to down-regulation of antigen processing and inhibition of the effector function of tumor-infiltrating lymphocytes [23].

Sobti et al. found high levels of protein markers associated with B cells, natural killer cells, macrophages, and regulatory T cells in cancer cells, opposed to the surrounding stroma. Likewise, they observed that biomarkers associated with suppressive populations of myeloid cells and exhausted T cells were numerous in peritumoral leukocytes, compared to in tumor cells [24]. Thus, therapeutic strategies have been proposed to target the tumor microenvironment by reactivating the antiviral immune response and subverting the immunosuppressive microenvironment [25,26,27]. For patients, immune checkpoint inhibitors, T cell therapy, and therapeutic Epstein–Barr Virus vaccines may be beneficial [28,29].

The rate of the immune cells dynamically changes with tumor progression, and the tumor-infiltrating lymphocyte levels reflect the treatment outcomes of the patients. Yoshizaki suggested that abundant intratumoral and stromal tumor-infiltrating lymphocytes are a predictive marker of favorable outcomes in patients with nasopharyngeal carcinoma [30].

Wang et al. evaluated the density and distribution of tumor-infiltrating lymphocytes in two independent cohorts and found that high tumor-infiltrating lymphocytes in the training set were significantly associated with favorable disease-free survival, overall survival, distant metastasis-free survival, and local-regional recurrent free survival [31].

Other cohort studies, as well as some large meta-analysis studies, indicate that tumor-infiltrating lymphocytes reflect the immunological heterogeneity of nasopharyngeal carcinoma and may represent a new prognostic biomarker [31,32,33,34,35]. Nasopharyngeal carcinomas with low levels of tumor-infiltrating lymphocytes are associated with poor overall survival and also poor disease-specific survival [32,33,34]. Moreover, an increased level of CD4-positive T cell infiltration was correlated with favorable overall survival, but the overall survival associated with CD3- and CD8-positive lymphocytes was not statistically significant [33]. In contrast, another meta-analysis study showed that tumor infiltration by CD3- and CD8-positive T cells is correlated positively with overall survival [35]. These results suggested that the role of tumor-infiltrating lymphocytes in mediating the antitumor immune response could be exploited in the treatment of patients with nasopharyngeal carcinoma [33,35].

There is no significant correlation between survival and tumor infiltration by CD68-positive or CD163-positive macrophages [35].

Nilsson et al. preformed a retrospective study and identified three specific immune phenotypes based on the presence and distribution of CD8-positive T cells: “inflamed”, “excluded”, and “deserted”, which carried important prognostic information and suggested that there was a difference in disease-free survival in favor of the “inflamed” over “excluded” type. The CD8-positive cells were identified in tumor cells, as well as in the surrounding stroma, and the antigen-presenting dendritic cells were observed largely in cancer cell areas. The dendritic cells have potential to facilitate the antigen cross-presentation necessary to execute cytotoxic T lymphocyte responses and therefore can be used for immunotherapy [36].

Furthermore, Sobti et al. found that in the “inflamed” phenotype, markers associated with B cells, natural killer cells, macrophages, and myeloid cells were highly expressed, while in the “excluded” phenotype, markers associated with suppressive populations of myeloid cells and T cells were more numerous in comparison to in the “inflamed” and “deserted” categories. The “desert” group had a higher level of granulocyte and immune regulatory markers. The study emphasizes the cellular composition of the nasopharyngeal carcinoma and could aid in stratifying patients for treatment based on their immune microenvironment [24].

CD45, CD4, CD8, CD20, and CD68 expression was also identified and quantified via immunofluorescence microscopy. CD4 was increased in the surrounding stromal leukocyte regions, and CD45, CD20, and CD68 were higher in immune-rich cancer cell islet regions [24].

Lu et al. identified, in the tumor microenvironment of nasopharyngeal carcinoma, nine types of inflammatory cells, including CD3-positive T lymphocytes, CD8 positive cytotoxic T lymphocytes, CD20 positive B lymphocytes, CD56 positive natural killer cells, FOXP3 positive regulatory T lymphocytes, CD1a-positive immature dendritic cells, CD83-positive mature dendritic cells, elastase-positive neutrophils and tryptase-positive mast cells. They found that the patients with a low density of tumor-infiltrating FOXP3 and CD8-positive T lymphocytes, neutrophils, and mast cells and the patients with a high density of natural killer cells showed a significantly longer overall survival and progression-free survival. It is suggested that the density of natural killer and mast cells could be used as biomarkers for predicting distant metastasis and recurrence in patients with nasopharyngeal carcinoma [37].

Information on antigen-presenting dendritic cells is limited. In a small series of untreated nasopharyngeal carcinomas, Nilsson identified, via multicolor flow cytometry, the main types of dendritic cells in the tumor immune microenvironment. A high frequency of CD1c-positive myeloid dendritic cells was observed and also the subset-specific expression of the C-type lectin receptor [38]. Another distinctive dendritic cell subtype marked by lysosome-associated membrane glycoprotein 3 has also been described in nasopharyngeal carcinoma. These dendritic cells lead to the downregulation of antigen processing and cause apoptosis suggesting that antigen-presenting dendritic cells can be also targeted for therapeutic purposes to facilitate the cross-presentation of antigens and aid in cell-mediated antitumor effects [22]. Dendritic-cell-based cancer therapy is a promising adjuvant therapy. It has been reported that there are increases i CD8- and CD4-positive T cells and the progression-free survival rate in dendritic cell immunotherapy patients compared to in the radiochemotherapy-treated patients [39,40].

Mast cells have an increased ratio and anti-tumor capacity in nasopharyngeal carcinoma, whereas in other malignant tumors, they have pro-tumorigenic potential. Hence, mast cells are correlated with a survival advantage in nasopharyngeal carcinoma [37,41].

Recurrent nasopharyngeal carcinoma has similar characteristics to primary carcinoma, although to a greater extent. The increased enrichment of immunosuppressive cells, particularly regulatory T cells, dendritic cells, and M2 polarized macrophages, and an even stronger effector exhaustion signal for tumor-infiltrating lymphocytes indicate a strong immunosuppressive microenvironment in both the primary and recurrent carcinoma [42,43].

Differences in certain characteristics of the tumor immune microenvironment were observed between Epstein–Barr-virus-positive and negative tumors. In Epstein–Barr-virus-positive tumors, genome sequencing revealed constitutive nuclear factor kappa beta activation, led by the viral expression of latent membrane protein 1 or somatic mutations. Downstream signaling persuades the increased secretion level of immunomodulatory molecules, such as interleukin-6, interleukin-8, and leukemic inhibitory factor, which eases the recruitment of immune cells to establish a chronic inflammatory environment [44,45]. Also, Jin et al. find high levels of the pro-inflammatory cytokine interferon gamma, which is produced in response to viral infection [43].

Epstein–Barr-virus-negative tumors show a tumor immune microenvironment with high levels of B lymphocytes, fewer regulatory T lymphocytes, and frequent M2 macrophages [46]. Epstein–Barr-virus-negative tumors also show increased intratumor heterogeneity, as seen by a higher level of cells and increased diversity within the tumor cells themselves [47].

## 6. Conclusions

The most common histopathological subtype of the nasopharynx is undifferentiated carcinoma, followed by the differentiated non-keratinized squamous cell carcinoma. Undifferentiated nasopharyngeal carcinoma had a male predominance, with a peak incidence in the 4th decade, while the differentiated subtype of non-keratinized squamous cell carcinoma had a peak incidence in females in the 6th decade.

For the epithelial malignant component, the immunohistochemical profile is relatively similar in the two histopathological subtypes. In the inflammatory cellular component, overlapping results were also observed; the CD20-positive B lymphocytes predominate, and the ratio of CD4:CD8-positive T cells was 1:1 in both subtypes, with cytotoxic T lymphocytes being more numerous intratumorally.

The tumor immune microenvironment is an important component of the large tumor microenvironment and plays a significant role in supporting tumor growth and progression. The knowledge of immune cellular components, as well as the understanding of the etiopathogenetic mechanisms intrinsic to the tumor process, represent a starting point for possible targeted therapies for nasopharyngeal carcinoma in the future.

## Figures and Tables

**Table 1:** Data related to the antibodies used for immunohistochemical reactions.

Antibody	Substrate	Clone	Dilution
CK AE1/AE3 ^1^	Mouse, Monoclonal	AE1/AE3	1:100
CK5 ^2^	Mouse, Monoclonal	XM26	1:100
p63	Mouse, Monoclonal	7JUL	1:25
CK7 ^3^	Mouse, Monoclonal	307M-94	1:100
CK20 ^4^	Mouse, Monoclonal	L26	1:150
34βE12 ^5^	Mouse, Monoclonal	34βE12	RTU ^6^
EMA ^7^	Mouse, Monoclonal	GP1.4	1:300
p53	Mouse, Monoclonal	DO-7	1:800
p16	Mouse, Monoclonal	CS1	1:150
EBV ^8^	Mouse, Monoclonal	CS1-4	RTU ^6^
LCA ^9^	Mouse, Monoclonal	X16/99	1:40
CD20 ^10^	Mouse, Monoclonal	L26	1:250
CD4 ^11^	Mouse, Monoclonal	4B12	1:100
CD8 ^12^	Mouse, Monoclonal	4B11	1:50
CD68 ^13^	Mouse, Monoclonal	514H12	1:100
CD1a ^14^	Mouse, Monoclonal	MTB1	RTU ^6^
CD117 ^15^	Mouse, Monoclonal	T595	1:20

^1^ CK AE1/AE3 (pan cytokeratin AE1/AE3); ^2^ CK5 (cytokeratin 5); ^3^ CK7 (cytokeratin 7); ^4^ CK20 (cytokeratin 20); ^5^ 34βE12 (high-molecular-weight cytokeratin); ^6^ RTU (ready-to-use); ^7^ EMA (epithelial membrane antigen); ^8^ EBV (Epstein–Barr virus); ^9^ LCA (leucocyte common antigen); ^10^ CD20 (cluster of differentiation 20); ^11^ CD4 (cluster of differentiation 4); ^12^ CD8 (cluster of differentiation 8); ^13^ CD68 (cluster of differentiation 68); ^14^ CD1a (cluster of differentiation 1a); ^15^ CD117 (cluster of differentiation 117).

**Table 2:** Signs and symptoms related to the presence of a tumor mass in the nasopharynx, Eustachian tube dysfunction and skull base and cranial nerve involvement.

Symptoms Due to the Presence of Nasopharynx Mass	Eustachian Tube Dysfunction	Skull Base and Cranial Nerves Involvement
Left nasal obstruction, oral respiration	Left mixed hearing loss	No
Bilateral nasal obstruction, oral respiration, hyposmia, snoring	Sensorineural bilateral hearing loss	Headache
Left nasal obstruction	Left conductive hearing loss, tinnitus	No
Left nasal obstruction, epistaxis	Left sensorineural hearing loss	No
No	No	No
Left nasal obstruction	Left sensorineural hearing loss	No
Left nasal obstruction	No	No
No	Right conductive hearing loss	No
Bilateral nasal obstruction	No	No
No	No	No
Bilateral nasal obstruction, oral respiration	No	Headache
Bilateral nasal obstruction	Left sensorineural hearing loss	Headache, diplopia
No	No	No
No	No	No
Bilateral nasal obstruction, oral respiration	Serous otitis media	No
Left nasal obstruction	Left mixed hearing loss	Headache
Right epistaxis	No	No
Bilateral nasal obstruction, snoring, dysphonia, cough	No	No
Right epistaxis	No	No
Left nasal obstruction, oral respiration	left conductive hearing loss, tinnitus	Headache
No	No	Headache
Bilateral nasal obstruction	Right conductive hearing loss	No
Left nasal obstruction, mucopurulent nasal discharge	No	Headache
Bilateral nasal obstruction, epistaxis	No	No
Bilateral nasal obstruction	Right conductive hearing loss	No
Bilateral nasal obstruction	No	Numbness, paresthesia
No	Left otalgia	No
Bilateral nasal obstruction, epistaxis	No	No
Left nasal obstruction, oral respiration	No	No
Bilateral nasal obstruction, oral respiration	No	No
No	No	No
Bilateral nasal obstruction, oral respiration, dysphonia	No	Nerve II paresis, deviation of the right eyeball
Left nasal obstruction, oral respiration	No	Headache
No	No	No
Bilateral nasal obstruction, oral respiration	Right otalgia, bilateral conductive hearing loss	No
Bilateral nasal obstruction, epistaxis	No	Headache

**Table 3:** The immunohistochemical profile of the nasopharyngeal undifferentiated carcinomas.

Antibody	Reaction Intensity	Malignant Squamous Component	Inflammatory Cellular Component	Cases/Rate of Positive Cases	Observations
AE1/AE3	+++ ^1^	100%	N/A ^5^	26/100%	Diffuse reaction
CK5	+++	100%	N/A	26/100%	Diffuse reaction
p63	+++	80–100%	N/A	26/100%	Diffuse reaction
34βE12	++ ^2^/+++	80–100%	N/A	25/96%	Diffuse reaction
EMA	++	10–80%	N/A	24/92%	Focal reaction
CK7	− ^3^	0%	N/A	0/0%	Positive intern control present
CK20	−	0%	N/A	0/0%	
p53	++/+++	80–100%	N/A	26/100%	Diffuse reaction
EBV	++/+++	80–100%	15%	2/8%	Diffuse reaction
p16	+++	100%	N/A	1/4%	Nuclear and cytoplasmic, intense, and diffuse reaction
CD117	+ ^4^/++	60–80%	0%	10/38%	Heterogeneous reaction, in malignant component, positive internal control present on mast cells
CD117	+++	0%	1–15%	20/80%	
LCA	+++	N/A	60–90%	26/100%	Peri- and intratumor
CD20	++/+++	N/A	70–85%	26/100%	Peri- and intratumor
CD4	++/+++	N/A	10–20% of lymphocytes	26/100%	Predominantly peritumoral
CD8	++/+++	N/A	5–10% of lymphocytes	26/100%	Predominantly peritumoral, CD4:CD8=1:1 in 75% of cases and 2:1 in 25% of cases
CD68	++	N/A	6–15%	23/88%	Macrophage and dendritic cells
CD1a	++	N/A	5–15%	23/88%	Predominantly intratumor dendritic cells, also CD68-positive

^1^ +++ intense reaction; ^2^ ++ moderate reaction; ^3^ − negative reaction; ^4^ + weak reaction; ^5^ N/A not applicable.

**Table 4:** The immunohistochemical profile of the differentiated keratinizing and non-keratinizing squamous cell carcinoma.

Antibody	Reaction Intensity	Malignant Squamous Component	Inflammatory Cellular Component	Cases/Rate of Positive Cases	Observations
CK AE1/AE3	+++ ^1^	100%	N/A ^2^	10/100%	Diffuse reaction
CK5	+++	100%	N/A	10/100%	Diffuse reaction
p63	+++	90–100%	N/A	10/100%	Diffuse reaction
34βE12	+ ^3^/++ ^4^	20–90%	N/A	10/100%	Focal/diffuse reaction
EMA	+/++	10–70%	N/A	10/100%	Focal/diffuse reaction
CK7	− ^5^	0%	N/A	0/0%	Positive intern control present
CK20	−	0%	N/A	0/0%	
p53	+/++	30–100%	N/A	10/100%	Focal/diffuse reaction
EBV	−	0%	N/A	0/0%	
p16	+++	90%	N/A	1/10%	Nuclear and cytoplasmic, intense and diffuse reaction
CD117	−	0%	0%	0/0%	Negative reaction in malignant component, positive internal control present on mast cells
CD117	+++	0%	1–10%	10/100%	Positive reaction on mast cells
LCA	+++	N/A	80–85%	10/100%	Peritumoral
CD20	++/+++	N/A	70–85%	26/100%	Peritumoral
CD4	++	N/A	10–20% of lymphocytes	10/100%	Peritumoral
CD8	++/+++	N/A	1–10% of lymphocytes	10/100%	CD4:CD8=1:1 in 90% of cases and 2:1 in 10% of cases
CD68	++	N/A	5–15%	10/100%	Dendritic cells, rare macrophages
CD1a	+++	N/A	5–15%	10/100%	CD68-positive dendritic cells

^1^ +++ intense reaction; ^2^ N/A not applicable; ^3^ + weak reaction; ^4^ ++ moderate reaction; ^5^ − negative reaction.

## Data Availability

The data that support the fundings of this study are available from the corresponding author upon reasonable request.
